# Spatiotemporal Dynamics of Insulitis in Human Type 1 Diabetes

**DOI:** 10.3389/fphys.2016.00633

**Published:** 2016-12-27

**Authors:** Kyle C. A. Wedgwood, Sarah J. Richardson, Noel G. Morgan, Krasimira Tsaneva-Atanasova

**Affiliations:** ^1^Centre for Biomedical Modelling and Analysis, University of ExeterExeter, UK; ^2^University of Exeter Medical School, University of ExeterExeter, UK; ^3^College for Engineering, Mathematics and Physical Sciences, University of ExeterExeter, UK; ^4^Engineering and Physical Sciences Research Council Centre for Predictive Modelling in Healthcare, University of ExeterExeter, UK

**Keywords:** type 1 diabetes, insulitis, agent-based modeling, spatio-temporal dynamics, peri-islet basement membrane

## Abstract

Type 1 diabetes (T1D) is an auto-immune disease characterized by the selective destruction of the insulin secreting beta cells in the pancreas during an inflammatory phase known as insulitis. Patients with T1D are typically dependent on the administration of externally provided insulin in order to manage blood glucose levels. Whilst technological developments have significantly improved both the life expectancy and quality of life of these patients, an understanding of the mechanisms of the disease remains elusive. Animal models, such as the NOD mouse model, have been widely used to probe the process of insulitis, but there exist very few data from humans studied at disease onset. In this manuscript, we employ data from human pancreases collected close to the onset of T1D and propose a spatio-temporal computational model for the progression of insulitis in human T1D, with particular focus on the mechanisms underlying the development of insulitis in pancreatic islets. This framework allows us to investigate how the time-course of insulitis progression is affected by altering key parameters, such as the number of the CD20+ B cells present in the inflammatory infiltrate, which has recently been proposed to influence the aggressiveness of the disease. Through the analysis of repeated simulations of our stochastic model, which track the number of beta cells within an islet, we find that increased numbers of B cells in the peri-islet space lead to faster destruction of the beta cells. We also find that the balance between the degradation and repair of the basement membrane surrounding the islet is a critical component in governing the overall destruction rate of the beta cells and their remaining number. Our model provides a framework for continued and improved spatio-temporal modeling of human T1D.

## 1. Introduction

Type 1 diabetes (T1D) is an auto-immune disease characterized by the selective destruction of pancreatic beta cells in the islets of Langerhans by the immune system (Eisenbarth, 1986; Atkinson, 2012; Boitard, 2012; La Torre and Lernmark, 2012; Pugliese, 2014; Richardson et al., 2014; Roep and Tree, 2014). This destruction takes place during an inflammatory phase, known as insulitis, in which various immune cells infiltrate the islets (Lecompte, 1958; Gepts, 1965; Willcox et al., 2009; Morgan et al., 2014). As the beta cell mass decreases over the course of the disease, the ability of the islets to secrete sufficient quantities of insulin to properly regulate blood glucose levels becomes compromised. As a result, patients with type 1 diabetes ultimately become reliant on the lifelong administration of external insulin.

Well-defined genetic components have been identified which predispose individuals to T1D; in particular the HLA-genotype (Itoh et al., 1993; Somoza et al., 1994). However, this alone is not sufficient to predict which individuals will develop the disease with precision. Since monozygotic twin studies have shown limited pairwise concordance for T1D (Barnett et al., 1981; Lo et al., 1991; Redondo et al., 2001), it is clear that environmental factors, such as viral infection, vitamin D status and childhood nutrition may also contribute significantly to the development of T1D (Knip et al., 2005).

Among the different subtypes of immune cells which infiltrate islets, CD8+ T cells are considered as the likely mediators of beta cell destruction (Bottazzo et al., 1985; Itoh et al., 1993; Somoza et al., 1994). It is widely believed that these promote beta cell apoptosis by both direct and indirect mechanisms and that macrophages then clear dying and dead beta cells very quickly. Other T lymphocytes, such as those expressing CD4, are also thought to play a role, though their precise functions are less clear (Willcox et al., 2009; Richardson et al., 2011). In addition to the T cells, B lymphocytes (CD20+) are also present in significant numbers during certain stages of insulitis in some patients. Indeed, recent evidence has suggested that the number of B cells and/or the ratio of B-cells to CD4+ cells present in the infiltrate can be used to classify the disease into two distinct phenotypes. These have been defined as “hyper-immune,” characterized by elevated numbers of CD20+ cells and a rapid loss of beta cell mass whereas, by contrast, the “pauci-immune” phenotype, is associated with a lower proportion of B cells and a much slower destruction of beta cells (Morgan et al., 2014; Leete et al., 2016). The mechanisms by which the B cells affect the rate of disease progression are unknown, but it is possible that they collaborate with CD8+ T cells to drive beta cell loss (Huppa and Davis, 2003).

Progress in understanding the cellular and molecular mechanisms underlying insulitis in humans has been hindered by the paucity of available samples from patients who died at, or close to, disease onset. Fewer than 200 such samples are available worldwide (Gepts, 1965; Foulis and Stewart, 1984; Klöppel et al., 1985; Dotta et al., 2007; Walker et al., 2011; Campbell-Thompson et al., 2012; Pugliese, 2014), and inferring the time course of a disease process from histological samples is fraught with difficulty since a range of assumptions and extrapolations about the likely progression are inevitably required to achieve this.

To offset this problem, much work has been performed using animal models such as the non-obese diabetic (NOD) mouse as a proxy for the human condition (Kachapati et al., 2012). The advantages of this are clear—experiments can be performed over relatively short periods of time and analysis of circulating and pancreatic lymphyocyte populations is achieved more readily during the course of disease. Such studies have, therefore, been extremely informative as a means to identify important cellular and molecular factors involved (Lally and Bone, 2003), although considerable effort needs to be made to verify that results are translatable to humans. While a large number of potential therapies have been identified in rodents, a means to prevent the human disease remains elusive (Brehm et al., 2012; In't Veld, 2014; Pugliese et al., 2014; Reed and Herold, 2015).

An alternative approach, which may shed light on human insulitis is offered by mathematical modeling. By constructing environments that mimic the pancreas and immune system, experiments can be performed *in silico*, and the results compared to empirical data arising from studies of relevant human tissue. Through model construction and analysis, candidate mechanisms giving rise to T1D can be interrogated in a systematic way. To this end, a number of authors have developed models describing: beta cell function (Bertram and Sherman, 2004), defective macrophage clearing (Marée et al., 2006), immune cell populations (Mahaffy and Edelstein-Keshet, 2007), multi-clonal populations of immune cells (Khadra et al., 2009, 2011; Jaberi-Douraki et al., 2015), immune cell cycles (Jaberi-Douraki et al., 2014b), and apoptotic stress generated by the loss of beta cell mass (Jaberi-Douraki et al., 2014c).

A common theme among the models outlined above is that they can be regarded as “lumped models,” in that they deal with averaged quantities taken over the whole body. For certain applications, this seems appropriate, as it is commensurate with current clinical practice. The biomarkers clinicians have available are typically derived from blood samples, in particular, measures of HbA1c and fasting glucose levels (American Diabetes Association, 2006; Inzucchi, 2012). Other biomarkers, such as measures of C-peptide (NIC, 2015) and islet cell and anti-insulin antibodies (Taplin and Barker, 2008) can be used to identify individuals who may be susceptible to diabetes and, though it is not currently routine clinical practice, they can also be used to aid classification of diabetes (Jones and Hattersley, 2013). Biomarkers obtained from blood samples are, by their nature, whole body measures. However, it is known from histological samples that insulitis displays a pronounced spatial dependence (Willcox et al., 2009; Morgan et al., 2014). This is due in part to the distribution of the islets within the pancreas, but is also a consequence of the presence of a basement membrane around the islets (Korpos et al., 2013), establishing a barrier to immune cell infiltration. Interactions and communication between immune cells also play a role and are likely to contribute to this spatial dependence.

Insulitis is a spatially heterogeneous process, both within an individual islet and across the pancreas as a whole. Islets that are heavily infiltrated can be located near to islets that are free from inflammation. Moreover, within an infiltrated islet, the destruction of beta cells appears not to follow a “wave-like” profile, as might be expected, but seems more random (Willcox et al., 2009; Morgan et al., 2014). These observations lend credence to the notion that immune cell communication is a critical component of insulitis.

To investigate insulitis and its relevance to the progression of T1D, these spatial aspects must thus be taken into account. Moreover, since the number of immune cells infiltrating any given islet is low (Willcox et al., 2009; Morgan et al., 2014), density based approaches, such as ordinary differential equation (ODE) modeling, cannot be applied with precision and alternatives are required.

In this manuscript, we construct an agent-based model of the insulitis process within a single islet. The model is posed on a regular domain that represents the space surrounding the islet. The principal agents in our model are the immune cells and we describe how they locate and target beta cells and how they interact with one another. By using an agent-based approach, we avoid the problems associated with density-based (averaged) descriptions of cell mass. We begin by introducing the relevant biology for our problem. Following this, we describe the development of, and results from, the agent-based model simulations. Finally, we conclude with a discussion of the model and its potential extensions.

## 2. Relevant biology

In this section, we highlight the biology corresponding to the components of our model.

### 2.1. Cell types

Our model consists of three distinct cell types—two types of lymphocyte and the beta cells. We consider in particular CD8+ T lymphocytes and CD20+ B lymphocytes. Of the various immune cells that have been shown to be involved in human insulitis, these are the ones that are believed to be most relevant to the questions under investigation in our study. The CD8+ cells are implicated in the destruction of the beta cells, while the CD20+ cells are deduced to play an important role, since their absence is associated with a weakly aggressive phenotype (Morgan et al., 2014). In our model, the B cells will essentially act as antigen presenting cells to the T cells (Rodríguez-Pinto, 2005). We assume that macrophages efficiently clear apoptotic beta cells. We do not account for mitosis in any of the cell types; whilst division and selection of relevant lymphocytes has been demonstrated in the lymphatic system, evidence for it in the peri-islet space is minimal (Willcox et al., 2010). Immune cells are assumed to have a finite lifespan and beta cells die following interactions with T cells (Cnop et al., 2010). We do not consider other cell types, such as α and δ cells, that are also found within the islets of Langerhans (Kim et al., 2009).

### 2.2. Basement membrane

Individual islets are contained within an encapsulating membrane. This membrane is comprised of various laminins and collagen (Korpos et al., 2013) and acts as barrier to invading immune cells. In mice with T1D, these compounds are lost, suggesting that the basement membrane has been degraded. In our model, we assume that the T cells are responsible for degrading the membrane, but in principle, other cells could be also responsible for this. We also make the assumption that the membrane can be repaired over time.

### 2.3. Cell movement

We assume that beta cells have a fixed location, but that immune cells are free to move around. The direction of this movement is mediated by a chemokine gradient, which will be described below (Stein and Nombela-Arrieta, 2005). In addition, immune cells of different types are attracted to one another, instigated by different chemical signaling pathways (Janeway et al., 1985). Cells are not allowed to pass through the basement membrane, nor through each other, but are allowed to remain in contact with one another.

### 2.4. Chemokine

The beta cells are considered to secrete a chemokine signal that attracts immune cells toward the islet (Christen and Von Herrath, 2004). In our study, we assume that the chemokine molecules are bound to the membrane of the beta cells and may also be cleaved off so that they diffuse freely.

### 2.5. Cell interactions

In their role as antigen presenting cells, the B cells can cause the T cells to enter an activated state, in which both their sensitivity to the chemokine gradient and their killing efficiency are enhanced (Friedl et al., 2005). We assume that activated T cells have a shorter lifespan than their non-activated counterparts (Green et al., 2003).

When a T cell encounters a beta cell, it triggers a pro-apoptotic pathway within the beta cell, ultimately resulting in the death of the beta cell (Cnop et al., 2005).

The behavior of the cells and membrane are summarized below.

### 2.6. Summary of basic behavior

Below, we will summarize the behavior of each of the cells types included in our modeling.

#### T cells

- Move up chemokine gradients
- Degrade basement membrane
- Kill beta cells
- Interact with B cells

When activated by B cells, T cells become more sensitive to the chemokine gradient and become more effective at killing beta cells.

#### B cells

- Move up chemokine gradients
- Form productive interactions with T cells
- Activate T cells

#### Beta cells

- Immobile
- Killed by T cells

We do not include in our model the possibility of beta cell replication. Studies have suggested that beta cell proliferation is increased, particularly in the early stages of insulitis (Willcox et al., 2010). Here, we disregard this effect.

#### Basement membrane

- Degraded by T cells.
- Can self-repair.

## 3. Methods

### 3.1. Building a cellular automaton

ODE-based modeling approaches average the dynamics associated with insulitis over space essentially representing the entire pancreas. Such approaches have provided numerous insights into the potential roles of T and B cell involvement in T1D and we refer the reader to Jaberi-Douraki et al. (2014a) for a recent and comprehensive review of this literature. However, we know that insulitis is heterogeneous and that the number of immune cells directly involved in the destruction of the beta cells in humans is low - far lower than would be necessary for a density approximation to be justified (Willcox et al., 2009; Morgan et al., 2014). Instead, we employ a different, agent-based, modeling approach (An et al., 2009; Holcombe et al., 2012). Our agents, the immune and beta cells, occupy finite area within a two dimensional space. In a real islet, cells are free to move in three dimensional space, but for ease of modeling (as a starting point of first approximation) and for a more direct comparison with data, we shall here restrict ourselves to the planar case. The agents are given specific rules to locomote, interact with one another and with the basement membrane. Simulations will then be performed to track emergent behavior arising from the basic rules. In this way, the model will evolve over a spatially extended domain in which cell-cell interactions can be explicitly defined.

Note that the rules that govern our agents' dynamics are not intended to be exhaustive lists for all potential behaviors exhibited by the cells. Instead, they are intended to represent a minimal set of interactions that can explain the observed phenomena. Additionally, the model can be iteratively developed to account for new interactions as required.

### 3.2. Cell movement

In this modeling framework, the cells are treated as being discrete circular particles (Levine et al., 2000; Palsson and Othmer, 2000; Tijskens et al., 2003; Maini and Baker, 2011; Bruna and Chapman, 2012; Plank and Simpson, 2012). Cell movement is simulated by modeling the forces that act upon cells from different sources and then by resolving these forces using Newton's second law of motion. In the absence of external forces, intrinsic forces acting upon the immune cells cause them to diffuse randomly, obeying a random walk. In the presence of a chemokine gradient, these same cells will seek to move up the gradient. Cells may be attracted to one another and may be in contact with one another for extended periods of time, that is, we allow cells to overlap, but they may not pass through each other.

### 3.3. Immune cell forces

Before discussing the governing equations, it is useful to define indexing sets for each of the cell types. In the following, we define the sets T,B, and **β** as sets containing the indices for T, B, and beta cells respectively. From Newton's second law of motion, we have:

(1) $$\begin{array}{l} m_{i}\frac{{\text{d}}^{2}x_{i}}{\text{d}t^{2}}=-\eta_{i}\frac{\text{d}x_{i}}{\text{d}t}+F_{i}+\sigma \xi_{i}\left(t\right),\;i\in T\cup B, \end{array}$$

for each cell where $x_{i}\in {\mathbb{R}}^{2}$ is the cell's location, *m*_*i*_ ∈ ℝ_≥0_ is the mass of the cell, η_*i*_ ∈ ℝ_≥0_ is the cell's viscosity and $F_{i}\in {\mathbb{R}}^{2}$ is the force acting on the cell. The final term in Equation (1) represents a Gaussian white noise process: 〈ξ(*t*)〉 = 0, 〈ξ(*t*)ξ(*t*+*s*)〉 = δ(*t – s*), with strength σ such that the immune cells perform random walks in the absence of any other forces (Palsson and Othmer, 2000; Middleton et al., 2014). In biological systems, we assume that the cells have low Reynold's numbers such that inertial forces are small compared to viscous ones. Under this approximation, we can replace (Equation 1) with:

$$\begin{array}{l} \frac{\text{d}x_{i}}{\text{d}t}=\frac{1}{\eta_{i}}F_{i}+\sigma \xi_{i}\left(t\right),\;i\in T\cup B \end{array}$$

For simplicity, we set η_*i*_ = 1 for all cells. Note that, in a general mathematical framework, specific values for η_*i*_ can be absorbed into the definition of *F*_*i*_. For our model definition, we include heterogeneity between immune cells through variations in their sensitivity to chemical gradients, as we shall discuss in Section 3.11. We will also initially assume that the cells within the subgroups are homogeneous with respect to their geometry, that is, they share a common radius *r*_imm_.

The force *F* can be broken up into its constituent parts:

(2) $$\begin{array}{l} F_{i}=F_{i}^{\text{chemo}}+F_{i}^{\text{cell-cell}}+F_{i}^{\text{islet}}+F_{i}^{\beta }. \end{array}$$

In the above, $F_{i}^{\text{chemo}}$ represents chemotactic force, $F_{i}^{\text{cell-cell}}$ represents cell attraction and repulsion, whilst $F_{i}^{\text{islet}}$ represents the interaction with the basement membrane and $F_{i}^{\beta }$ represents interaction with the beta cells.

### 3.4. Chemokine signaling

Since the chemokine is a chemical signal, we establish a gradient using a reaction-diffusion equation:

(3) $$\begin{array}{l} \frac{\partial C}{\partial t}=D\nabla^{2}C+f\left(C\right). \end{array}$$

We assume that the chemokine evolves on a much faster timescale compared to the movement of the cells, so we assume the chemokine to be at steady state by setting the LHS of Equation (3) to zero. The domain on which we simulate our model represents only a small space around an individual islet. We note that this region is small compared to the entire pancreas, and further that the chemokine is free to diffuse out of our prescribed region. Typically, when solving PDEs, boundary conditions are applied at the edge of the domain over which the equation is being solved. However, as we are treating our region as only a small part of a much larger one, we feel it is more appropriate to not apply boundary conditions, leaving them open. The reaction term is given by local decay with point sources given by the locations of the beta cells. The chemokine equation now reads:

(4) $$\begin{array}{l} D\nabla^{2}C=\lambda C-\underset{j\in \beta }{\sum }\nu_{j}\left(t\right)\delta \left(x-x_{j}\right), \end{array}$$

where λ is the degradation rate of the chemokine and *x*_*j*_ are the centroids of the beta cells. To reflect the fact that dead beta cells will not secrete chemokine, we assume that the production of chemokine is dependent on the current viability of the cell ν_*j*_(*t*). For a viable beta cell, ν = 1, whilst dead beta cells have ν = 0. This equation can itself be recast as:

(5) $$\begin{array}{l} QC=\underset{j\in \beta }{\sum }\nu_{j}\left(t\right)\delta \left(x-x_{j}\right),\;Q=\left(\lambda -D\nabla^{2}\right). \end{array}$$

The Green's function for the operator *Q* at a given time is a Gaussian function and so we lump parameters together and choose a form for the chemokine signal given by:

(6) $$\begin{array}{l} C\left(x,t\right)=\underset{j\in \beta }{\sum }\alpha \nu_{j}\left(t\right)\exp\left(-{\left(x-x_{j}\right)}^{2}/\left(2\sigma_{C}^{2}\right)\right), \end{array}$$

where α now represents the strength of the chemokine signal and σ_*C*_ represents the range over which it decays. In order to represent our assumption that the chemokine exists in forms in which it is bound to the membrane and forms in which it freely diffuses, we replace Equation (6) by:

(7) $$C(x,t)=\sum_{k\;=1}^{2}\sum_{j\in \beta }\alpha \nu_{j}(t)C_{0}^{k}\exp\left(-{\left(x-x_{j}\right)}^{2}/(2\sigma_{C,k}^{2})\right),$$

where σ_*C*, 1_ is small and reflects the membrane bound chemokine, whilst $C_{0}^{k},k=1,2$ control the proportion of chemokine that is membrane bound vs. that which is freely diffusing. The immune cells respond to this gradient via:

$$F_{i}^{\text{chemo}}=S_{i}\nabla C,$$

where *S*_*i*_ is the sensitivity of the *i*'th cell to the chemokine gradient. We note that this represents the notion that immune cells tend to move up a potential gradient in which the locations of the beta cells are given by local peaks of this potential. The use of such potentials to model forces is an integral part of this modeling framework (Middleton et al., 2014).

### 3.5. Cell-cell interactions

We assume that T and B cells are attracted to one another. For simplicity's sake, we assume that this can be modeled via another Gaussian function. It is worth noting that this could represent attraction acting at a distance, or could represent a local effect acting to keep cells in contact with one another (or some combination of the two). We do not allow cells to pass through one another. We can achieve both of these through the use of another potential. Common choices for these potentials include the Lennard–Jones potential and the Morse potential (Middleton et al., 2014). We shall use the following potential:

(8) $$\begin{array}{l} U_{i}^{\text{cell-cell}}=\sum_{j\neq i,j\in T\cup ℬ}A_{\text{a}}^{ij}\exp(-|x_{j}-x_{i}{|}^{2}/d_{\text{a}}^{2})\\ \;-\;A_{\text{r}}{\left(|x_{j}-x_{i}|-2r_{\text{imm}}\right)}^{6}H(|x_{j}-x_{i}|-2r_{\text{imm}}). \end{array}$$

The first of these terms is the attraction between immune cells of different types—B cells seek out T cells to activate and T cells are also attracted to B cells. This is achieved by setting:

$$A_{\text{a}}^{ij}=\{\begin{array}{ll} A_{\text{a}}^{0} & \chi_{i}\neq \chi_{j}\\ 0 & \chi_{i}=\chi_{j} \end{array},$$

where χ_*i*_ is an indicator function taking value 1 if cell *i* is a T cell and 0 if it is B cell. The spatial scale of this attraction is set by *d*_*a*_.

The second term accounts for the fact that cells cannot pass through one another. If the distance between cells is greater than the sum of their radii, that being 2*r*_imm_, they exert no repulsive force on one another, achieved through the inclusion of a Heaviside function *H*. However, if this distance falls below this sum, a large repulsive force is exerted. This is known as a hard-core potential (Levine et al., 2000). Note that some authors choose this potential to be infinite at the point of contact, so that cells cannot occupy or share any part of physical space (Bruna and Chapman, 2012). However, this necessitates choosing a smaller time step and, since cells can remain in contact with one another for significant periods of time, we will not make such a choice. Note also that the parameter *A*_*r*_ is shared amongst all immune cells. Once again, the forces acting on the *i*'th cell can then be expressed as:

(9) $$\begin{array}{l} F_{i}^{\text{cell-cell}}=-\nabla U_{i}^{\text{cell-cell}}. \end{array}$$

Note that, since immune cells move around the domain, the potential “landscape,” together with its peaks and troughs, is constantly evolving.

### 3.6. Islet interactions

For simplicity, and to remain consistent with our chosen geometry of the cells, the basement membrane encapsulating the islet is chosen to be a circle centred at the origin with radius *R*. Cells cannot pass through the membrane, so when it is present, immune cells remain on the inside or outside of the islet. This can be reflected through the use of another hard-core potential. Whilst we have not yet defined the dynamics for the membrane, we shall assume that there is a integrity threshold, *h*, below which the membrane does not prevent cells from passing through. Since we do not expect the membrane to have the same integrity across its whole length, we also need to account for the location at which the cell interacts with the islet. The relevant hard-core potential is then given by:

(10) $$\begin{array}{l} U_{i}^{\text{islet}}=\;A_{\text{islet}}H(m(\theta )-h)H(r_{\text{imm}}-|d_{i}-R|)\\ \;{\left(r_{\text{imm}}-|d_{i}-R|\right)}^{6},\\ \;d_{i}=\;|x_{i}-x_{\text{islet}}|, \end{array}$$

where *m*(θ) ∈ [0, 1] is the membrane integrity at angle $\theta \in S^{1}$ around the membrane, $x_{\text{islet}}\in {\mathbb{R}}^{2}$ is the location of the center of the islet and *A*_islet_ is a constant indicating the strength of repulsion. Once more, the force acting on the cells is then given by:

(11) $$\begin{array}{l} F_{i}^{\text{islet}}=-\nabla U_{i}^{\text{islet}}. \end{array}$$

Finally, we must define the forces acting upon the immune cells exerted by the beta cells. This is functionally the same as the repulsive force between cells (Equation 8) and is given by:

(12) $$\begin{array}{l} U_{i}^{\beta }=A_{\text{r}}\sum_{j\in \beta }\nu_{j}(t)H(|x_{j}-x_{i}|-(r_{\beta }+r_{\text{imm}}))\\ \;{\left(\left|x_{j}-x_{i}|-(r_{\beta }+r_{\text{imm}}\right)\right)}^{6}, \end{array}$$

where *r*_β_ is the radius of the beta cells. The time-dependence of ν_*j*_ here reflects that the beta cells may die, after which we no longer need to consider repulsive effects generated by them (assuming the dead cell bodies are cleared by macrophages), and so ν_*j*_ is set to 0 for that cell. The final force in Equation (2) is defined through

(13) $$\begin{array}{l} F_{i}^{\beta }=-\nabla U_{i}^{\beta }. \end{array}$$

### 3.7. Immune cell lifespan

The timescale over which insulitis takes place is long compared to the average lifespan of an immune cell. Thus, we need to incorporate immune cell death into our model. We assume that T and B cells have a lifespan of *L*_T_, and *L*_B_ respectively. Each cell then has a counter χ_*i*_, which is incremented by setting χ_*i*_ ↦ χ_*i*_ + 1 at each time step. Activated T cells are expected to have shorter lifespans than unactivated T cells (Green et al., 2003), so for those cells, we instead update the counter via χ_*i*_ ↦ χ_*i*_ + Δχ where Δχ > 1.

When χ_*i*_ exceeds *L*_T_ (or *L*_B_) for a given T (B) cell, it is considered to be dead and is removed. We keep the number of T and B cells constant throughout the simulation by assuming that each dead cell is replaced by a newly arriving one. We assume that the vasculature is sufficiently dense that immune cells can enter at any point in the extra-islet space. As such, the location of the new cell is drawn randomly from a uniform distribution over the extra-islet space, achieved through the use of polar coordinates. The cell's counter is reset to 0 and, if the cell is a T cell, it is chosen to be in the unactivated state.

### 3.8. Membrane dynamics

In our simplified geometry, the encapsulating membrane is a represented by a circle, which can be parameterized by a single variable $\theta \in S^{1}$. The equation governing the evolution of the membrane viability, *m*, is given by:

(14) $$\begin{array}{l} \frac{\text{d}m(\theta )}{\text{d}t}=\alpha_{m}(1-m(\theta ))\\ \;-\lambda_{m}\sum_{i\in T}\exp\left(-(|x_{i}-x_{m}(\theta )|-r_{\text{imm}})/d_{m}\right), \end{array}$$

where α_*m*_ and λ_*m*_ are respectively the repair and degradation rates of the membrane and *x*_*m*_(θ) is the location in real space of the membrane at position θ. The term in the sum represents the fact that we expect T cells to break down the membrane and this can only occur when T cells are close enough. The range over which the T cells can degrade the membrane is set by *d*_*m*_.

We note that as beta cells are destroyed, the morphology of the affected islets may change. In particular, some islets in T1D present with small size and irregular outline (Gepts, 1965). The membrane in our study, where it exists, simply follows a circle with fixed radius, and thus does not account for these changes. However, we note that not all degranulated islets have small size and irregular outline (Gepts, 1965). Moreover, the morphology of islets, irrespective of insulitis, is highly variable. It is thus difficult to infer how the morphology of a given islet varies over the course of the insulitic process. Nevertheless, it seems likely that changes to islet morphology will occur, and we discuss approaches to incorporate this into our model in Section 5.1.1.

### 3.9. Activation and apoptosis

When T cells and B cells are in contact with one another, we assume that B cells can activate non-activated T cells and that this process has a characteristic time course. We thus describe the activation level of the *i*'th T cell, *a*_*i*_, via:

(15) $$\frac{\text{d}a_{i}}{\text{d}t}=\{\begin{array}{ll} \begin{array}{l} \sum_{j\in ℬ}H(|x_{j}-x_{i}-2r_{\text{imm}}+0.1|)\\ \;-\lambda_{a}a_{i} \end{array} & a_{i}<1\\ 0 & a_{i}\geq 1, \end{array},i\in T,$$

where λ_*a*_ is the decay rate of the activation signal. The constant 0.1 is included inside the Heaviside function to account for the fact that we are not explicitly modeling cell-cell contacts, and the use of hard-core potentials tends to make cells move apart quickly when they are close. Our specific choice for this constant is selected such that cells are allowed to remain in contact with one another. We note that if this value is selected to be too large, cells can essentially occupy the same location, whilst values that are too small will cause cells to “bounce” off one another. We wish to avoid both of these behaviors, and over a range of choices, we found that a value of 0.1 satisfied these criteria. Once the activation signal reaches 1, the cell is activated and remains so until that cell dies, (or until the end of the simulation if that occurs first), reflected by setting the RHS to zero upon the activation reaching 1. Activated cells have an increased sensitivity to the chemokine signal and enhanced killing rate. This is captured in the model by increasing the sensitivity and killing parameters:

$$S_{i}\mapsto S_{i}+\Delta S,\;\kappa_{i}\mapsto \kappa_{i}+\Delta \kappa ,$$

where Δ*S* and Δκ are positive constants.

The dynamics for the apoptosis of beta cells *b*_*i*_ follows a similar prescription:

$$\frac{\text{d}b_{i}}{\text{d}t}=\{\begin{array}{ll} \begin{array}{l} \;\sum_{j\in T}\kappa_{j}H(|x_{j}-x_{i}-2r_{\text{imm}}+0.1|)\\ \;-\lambda_{b}b_{i} \end{array} & b_{i}<1\\ \;0 & b_{i}\geq 1, \end{array},i\in \beta ,$$

where λ_*b*_ is the decay rate of the apoptotic signal, κ_*j*_ is the killing rate of the *j*'th T cell, and we include the constant, 0.1, for the same reasons as for (Equation 15). If *b*_*i*_ exceeds 1 for a given cell *i*, that cell is assumed to have been killed and is removed from the simulation. This is achieved by setting its viability, ν_*i*_, to be zero.

### 3.10. Implementation

Since the system we are solving is a Langevin equation, we must use an appropriate numerical method to deal with the stochasticity. For computational efficiency, we shall use the forward Euler–Maruyama scheme. This method only has strong order dt^1/2^ but requires the fewest function evaluations of any of the solvers for stochastic differential equations (SDEs). The one exception to this is the equation governing the membrane dynamics where the explicit Euler scheme is unstable and causes solutions to blow up. For this equation, we use an implicit, backward Euler scheme. This keeps the total order accuracy of solutions consistent with the other equations, but provides the required stability without incurring additional computational cost.

Note that the membrane (Equation 14) treats distinct points along the membrane as being distinct from one another. This is the only system that is grid-based – all of the other equations are grid-free. In order to solve this system, we first discretize the membrane into *N*_θ_ points, θ_*i*_ = − π + 2π(*i* − 1)/*N*_θ_, *i* = 1, *N*, and subsequently solve (Equation 14) at each of these points. To find the value of the *m* at a point not on the discrete grid, we use band-limited interpolation for periodic signals as described in Schanze (1995).

### 3.11. Initial conditions and parameters

**Figure 1:**
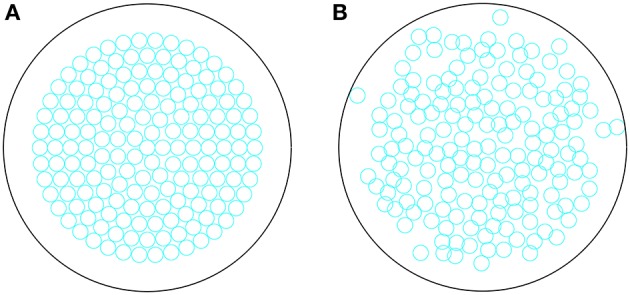
**(A)** Initial configuration of beta cells within an islet, distributed in concentric circles. **(B)** Final configuration of the beta cells evolved for *T* = 1000 timesteps, according to Equations (16–18).

The immune cells are initially located at random, non-overlapping positions within annular domain with inner radius *R* and outer radius 1mm. The islet is positioned at the center of the domain, which for simplicity, we set as our origin. To distribute the beta cells, *N*_β_ cells are arranged in concentric circles within the islet. Next, for *T* = 1000 timesteps, they are allowed to freely evolve governed by:

(16) $$\begin{array}{l} \frac{\text{d}x_{i}}{\text{d}t}=F_{i}^{\text{islet}}+F_{i}^{\beta -\beta }+\sigma \xi_{i}\left(t\right),\;i\in \beta , \end{array}$$

where *x*_*i*_ is the center of the *i*'th beta cell, $F_{i}^{\text{islet}}$ is the same as Equation (13) operating on the beta cells rather than the immune cells with:

(17) $$\begin{array}{l} F_{i}^{\beta -\beta }=-\nabla U_{i}^{\beta -\beta }, \end{array}$$

where

(18) $$\begin{array}{l} U_{i}^{\beta -\beta }=\underset{j\neq i,j\in \beta }{\sum }H\left(|x_{j}-x_{i}|-2r_{\beta }\right){\left(|x_{j}-x_{i}|-2r_{\beta }\right)}^{6}, \end{array}$$

where $x_{i}\in {\mathbb{R}}^{2}$ now represents the location of the beta cell. After the *n* steps, the beta cells are fixed in location for the remainder of the simulation. Note that during this process, the immune cells are fixed in position. We distribute cells in this manner as it (a) generates a more realistic islet geometry compared with spacing them evenly within the islet and (b) it overcomes the known computational problems with randomly distributing non-overlapping disks in a confined region (Song et al., 2008). In Figure 1, we show the initial and final configuration of the beta cells following this approach. All simulations and analyses were performed in Matlab.

Other initial conditions are given by *m* = 1 for all θ, *a*_*i*_ = 0 for all i∈T, and *b*_*i*_ = 0, ν_*i*_ = 1, ∀*i* ∈ β.

The sensitivities of the T and B cells to the chemokine gradient are drawn from distinct Gaussian distribution with means *S*_*T*_ and *S*_*B*_ and variances σ_*T*_ and σ_*B*_ respectively. Parameters governing the geometry of our domain, such as immune cell and beta cell sizes were chosen to be matched to available data. In particular, beta cell diameters were taken from Saisho et al. (2013), immune cell diameters were based on values found in Wang et al. (2012), numbers of cells in each immune cell population were taken from Willcox et al. (2009) and islet composition (in terms of beta cells) were based on those found for humans in Kim et al. (2009). Data regarding the *in vivo* lifespan of specific effector lymphocytes in humans are rare and these may also be heterogeneous even within the same population. However, effector lymphocytes are unlikely to have lifespans ranging beyond several weeks (Sprent, 1993). To address the uncertainty in lifespans, we performed simulations across a range of values, from 3 to 7 weeks and compared results.

Other parameters in our model were tuned to provide the required behaviour, though we comment that much of the general behavior observed in our simulations is robust to parameter variations, suggesting that the model outcomes are robust. We note that certain processes, such as the degradation and repair of the peri-islet basement membrane, are phenomenological in nature, and as such, it is difficult to ascribe to them meaningful parameter values. Whilst the islet membrane is a physical component of the real system, we represents its viability by a scalar in the range 0–1, and so parameter values should be interpreted with respect to this scaling. Similar comments hold for the chemokine signal. Though a chemoattractant gradient is thought to exist in the peri-islet space, it is not clear what its composition, and subsequent properties, might be. As such, we have chosen a prototypical form to represent our chemokine signal, and investigated how changes to the strength of this signal impacts upon the resulting dynamics. All parameter values are summarized in Table 1 and referenced in text where they are altered for specific numerical experiments.

**Table 1:** **Table of parameter values and meanings for the agent-based model**.

*N*_*T*_	Number of T cells	30 Ref: Willcox et al. (2009)
*N*_*B*_	Number of B cells	{5, 30} Ref: Willcox et al. (2009); Leete et al. (2016)
*N*_β_	Number of beta cells	166 Ref: Kim et al. (2009)
σ	Strength of Wiener process	1.0 μm days^−1^
*r*_imm_	Radius of immune cells	4.0 μm Ref: Wang et al. (2012)
*r*_β_	Radius of beta cells	6.4 μm Ref: Saisho et al. (2013)
*R*	Radius of islet	120.0 μm Ref: Kim et al. (2009)
*S*_*T*_	Mean sensitivity of T cells	600 N^−8^ μm mM^−1^
*S*_*B*_	Mean sensitivity of B cells	700 N^−8^ μm mM^−1^
σ_*T*_	Variance of sensitivity of T cells	0.5 N^−8^ μm mM^−1^
σ_*B*_	Variance of sensitivity of B cells	0.5 N^−8^ μm mM^−1^
$\bar{\kappa }$	Killing rate	0.2 cell^−1^ day^−1^
α_*C*, 1_	Chemokine strength	0.5 μM
σ_*C*, 1_	Range of chemokine signal	200.0 μm
α_*C*, 2_	Chemokine strength	10.0 μM
σ_*C*, 2_	Range of chemokine signal	8.0 μm
$A_{a}^{0}$	Attraction strength between T and B cells	1.0 N^−8^ μm
*d*_*a*_	Attraction range between T and B cells	4.0 μm
*A*_*r*_	Repulsion rate for hard-core potential between cells	10.0 N^−8^ μm
*A*_islet_	Repulsion rate for hard-core potential of islet	100.0 μm
α_*m*_	Repair rate of membrane	0.01 day^−1^
λ_*m*_	Degradation rate of membrane	0.1 cell^−1^ day^−1^
*d*_*m*_	Membrane degradation range	4.0 μm
λ_*a*_	Decay rate of activation signal	0.5 day^−1^
λ_*b*_	Decay rate of apoptotic signal	0.5 day^−1^
*ΔS*	Activated sensitivity boost	400 N^−8^ μm mM^−1^
*Δκ*	Activated killing rate boost	0.9 cell^−1^ day^−1^
*L*_T_	Lifespan of T cells	{21, 28, 56} days Ref: Sprent (1993)
*L*_B_	Lifespan of B cells	{21, 28, 56} days Ref: Sprent (1993)
*Δκ*	Activated killing rate boost	0.9 cell^−1^ day^−1^
*Δχ*	Activated cell lifespan counter	2
dt	Time-step for EM scheme	0.001 day^−1^

*Where indicated, references show the studies upon which specific parameter values were based. Parameters are altered as discussed in the text for specific numerical experiments*.

## 4. Results

**Figure 2:**
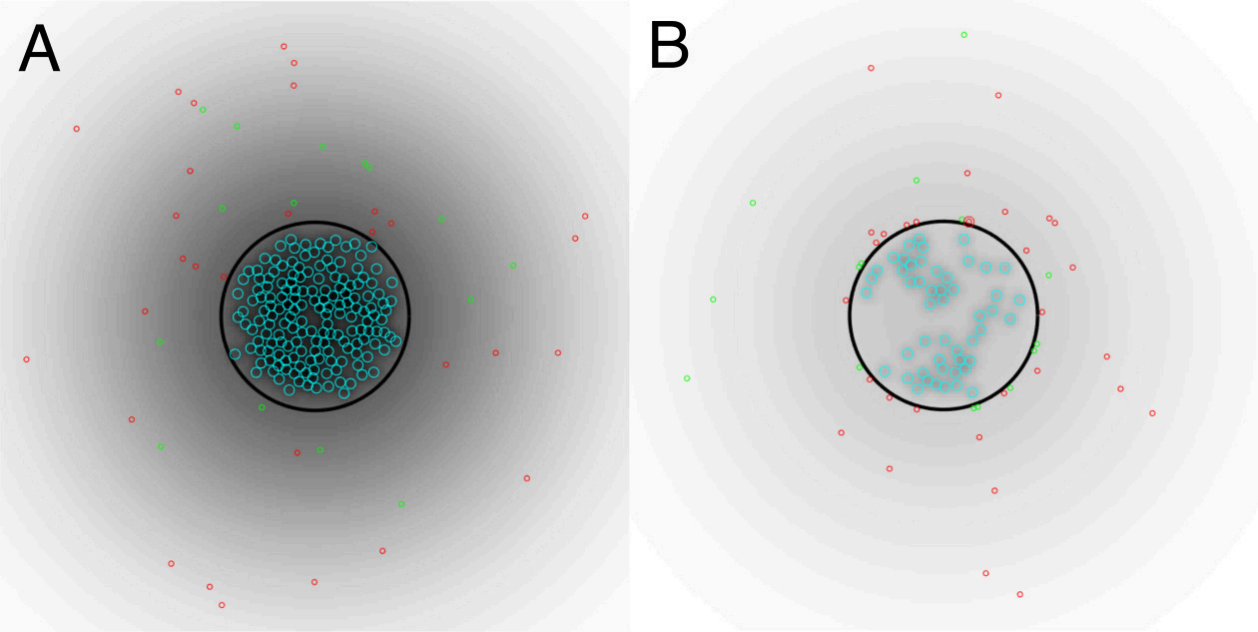
**(A)** Initial state of system. **(B)** State of the system after *T* = 4000 days. A video of the full time-course of this simulation can be found in the Supplementary Material. In both panels, the blue circles represent beta cells, the red circles represent T cells, the green circles represent B cells, the black circle represents the peri-islet basement membrane and the black gradient represents a chemokine signal. Parameters are as in Table 1.

**Figure 3:**
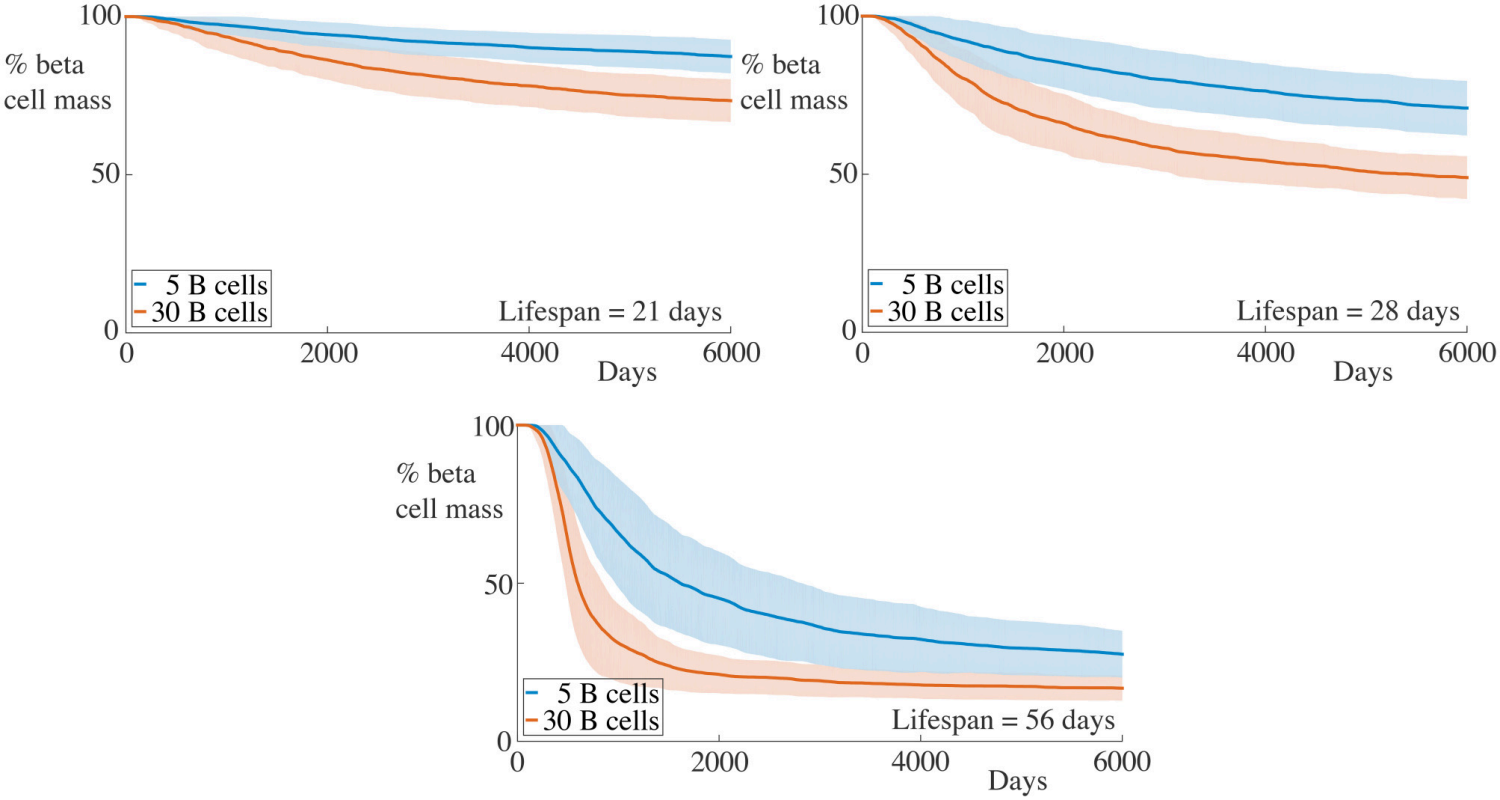
**Effect of variation of B cell number on beta cell mass loss in the agent-based model**. Each graph shows the mean ± SEM for 100 simulations. Across all immune cell lifespans, we find that increasing the number of B cells significantly increases the destruction rate of the beta cells. Parameters are as in Table 1. Videos of typical simulations for the case with immune cell lifespans of 21 days may be found in Videos S1, S2.

**Figure 4:**
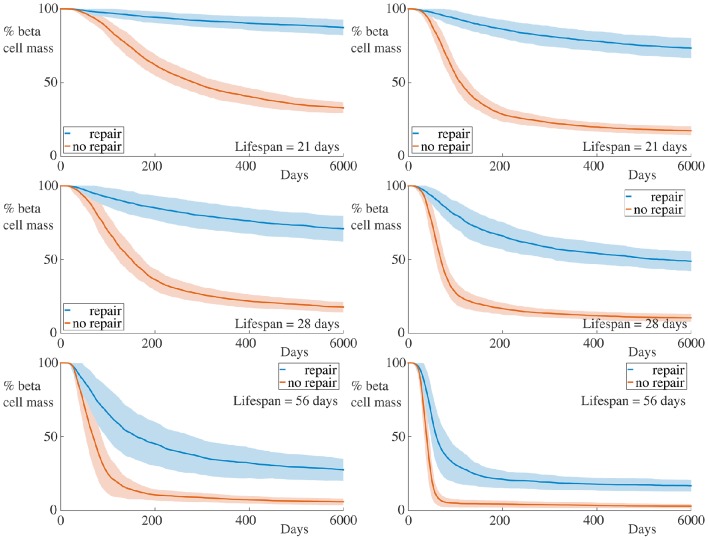
**Time-course of beta cell mass comparing conditions with and without peri-islet basement repair**. Each graph shows the mean ± SEM for 100 simulations. Data for simulations with 5 B cells are displayed in the left column, whilst those for simulations with 30 B cells are depicted in the right column. In the case without membrane repair, we observe both an increase in the rate of beta cell destruction and pronounced differences in the final beta cell mass across all immune cell lifespans and B cell number. Parameters are as in Table 1 with α_*m*_ = 0. Videos of typical simulations for the case with immune cell lifespans of 21 days may be found in Videos S3, S4.

**Figure 5:**
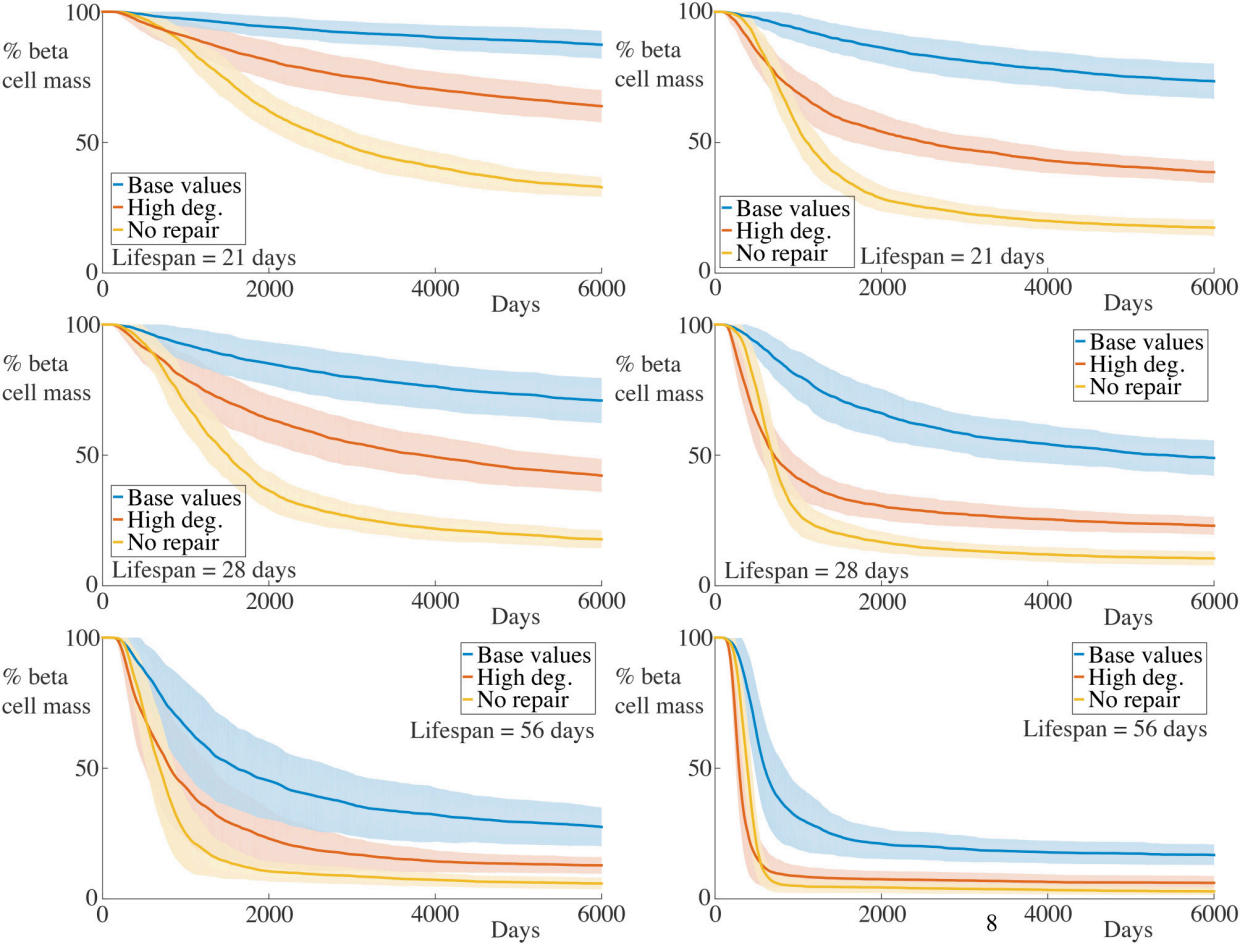
**Time-course of beta cell mass comparing with higher membrane degradation rates**. Each graph shows the mean ± SEM for 100 simulations. The left column shows results for simulations with 5 B cells whilst the right column shows those with 30 B cells. Data for the condition with the base value for membrane degradation but with α_*m*_ = 0, as reported in Figure 4 are also shown. An increase in the beta cell destruction rate, as well as lower final beta cell masses are observed across all immune cell lifespans and number of B cells. Note, however, that whilst the initial rate of beta cell destruction for the condition with high membrane degradation is higher than that for the case with no membrane repair, the final beta cell mass is higher for the former compared with the latter. We refer the reader to the text in Section 4.3 for further interpretation of this observation. Parameters are as in Table 1 with λ_*m*_ = 0.2 for the high degradation condition and α_*m*_ = 0 for the no membrane repair condition. Videos of typical simulations for the case with immune cell lifespans of 21 days may be found in Videos S5, S6.

**Figure 6:**
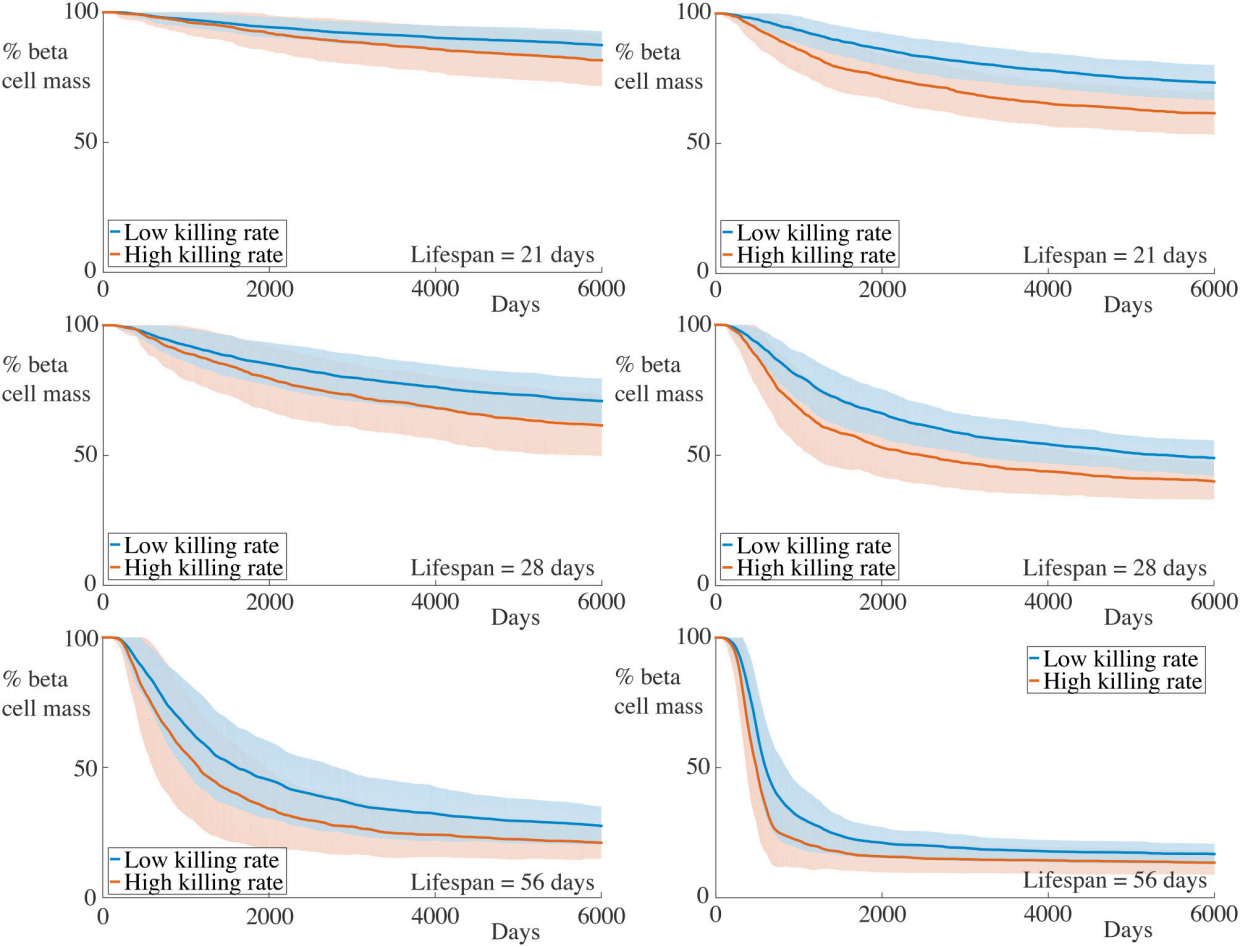
**Time-course of beta cell mass comparing with higher activated killing rates**. Each graph shows the mean ± SEM for 100 simulations. The left column shows results for simulations with 5 B cells whilst the right column shows those with 30 B cells. Although an increase in the rate of beta cell destruction is observed, this is relatively small in all cases. Parameters are as in Table 1 with Δκ = 1.8.

**Figure 7:**
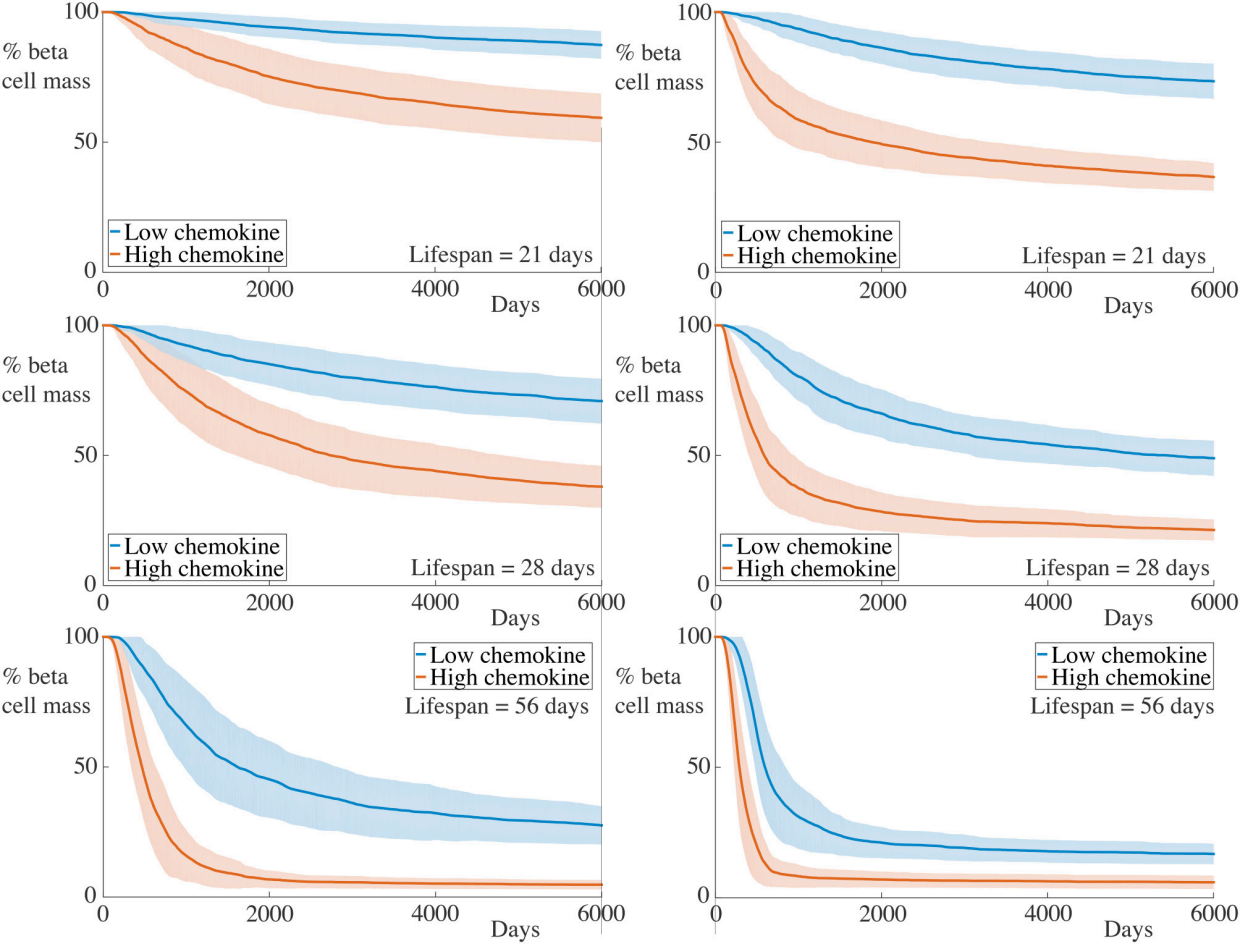
**Time-course of beta cell mass comparing conditions with the base and increased strength of chemokine signal**. Each graph shows the mean ± SEM for 100 simulations. The left column shows results for simulations with 5 B cells whilst the right column shows those with 30 B cells. In the high chemokine signal parameter regime, we observe both an increase in the rate of beta cell destruction and pronounced differences in the final beta cell mass across all immune cell lifespans and B cell number. These differences are greater than those displayed for higher T cell killing rates in this figure. Parameters are as in Table 1 with $\alpha_{C}^{1}=1.0$ and $\alpha_{C}^{2}=20.0$. Videos of typical simulations for the case with immune cell lifespans of 21 days may be found in Videos S7, S8.

In Figure 2, we show the initial state of a prototypical simulation, and the state of the system at *T* = 4000 days. Here, we can see that the islet exhibits significant beta cell loss. We also see that immune cells congregate at the islet membrane and that a small number infiltrate the islet itself. Across all parameter sets used in our experiments, this behavior is preserved. In the Supplementary Material, we provide videos showing the full time-course of typical simulations with base parameters as indicated in Table 1, lymphocyte lifespan of 21 days and other parameters selected to reflect a variety of conditions, as discussed in the forthcoming sections. These simulations may be compared with the averaged results shown in Figures 3–**7**.

For all parameter sets, we simulate 100 realizations and average at specific time points to develop a time-course. During the simulations, we keep track of the number of immune cells within the islet and the number of viable beta cells. We take the total area encompassing the viable beta cells as being reflective of the beta cell mass. We shall now report both the time-courses of the remaining beta cell mass as a percentage of the initial mass (calculated as the ratio of the total mass of viable beta cells at time *t* and the initial mass of beta cells, averaged over the 100 realizations), and the number of immune cells in the islet for a number of conditions, along with the standard error of the mean (SEM) at each time point. In each case, we shall use the base set of parameters as indicated in Table 1, changing specific values where indicated. In all cases, to assess how the lifespan of the immune cells affects the resulting dynamics, we vary this between 21, 28, and 56 days as indicated in the figures.

### 4.1. Varying number of B cells

We begin by examining a key condition in this study, namely how the number of B cells in the peri-islet space impacts the rate of destruction of beta cells. To address this, we compare a condition with few B cells, setting *N*_B_ = 5, and one with a higher number of B cells, with *N*_B_ = 30. These results are summarized in Figure 3.

We observe that when the immune cell lifespan is short, neither the low nor the high B cell condition exhibit significant beta cell destruction. As the lifespan is increased, we observe a pronounced increase in this rate. For an immune cell lifespan of 56 days, we see that the high B cell condition reaches an equilibrium value at around 3000 days. Note that the equilibrium value is below the critical threshold of ~20% of remaining beta cell mass (Cnop et al., 2005) (however, it should be noted that this threshold is a whole-body threshold and may not be valid for an individual islet).

### 4.2. Varying repair rate of membrane

We have assumed in our model that the peri-islet basement membrane is capable of being repaired at a rate α_*m*_. If we decrease α_*m*_, the rate of degradation of the membrane may be such that holes caused by infiltrating T cells are not repaired (at least over the timescale of our simulation). In the extreme case, the membrane is incapable of repair, in which case we set α_*m*_ = 0. In Figure 4, we display the time-course of a simulation in such a regime.

It is clear from these graphs that removing the repair mechanism of the membrane dramatically increase the susceptibility of the beta cells to cytotoxicity. In the most extreme case, in the situation with few B cells and a short immune cell lifespan, we now see a huge difference in both the rate of beta cell destruction and the final cell mass at the end of our simulation. In the case with no membrane repair, the final beta cell mass of almost all simulations is below ~20% of the initial mass. These results highlight the importance of the peri-islet basement membrane to the progression of insulitis, and also suggest that ongoing repair of this membrane is critical to slowing the infiltration of the immune cells.

We also observe, in contrast to the results in Figure 3, that the immune cell lifespan now has a more muted affect on the resulting dynamics. Whilst there is an increase in the rate of beta cell destruction with increasing lifespan, the more significant contribution appears to be to lower the equilibrium value of remaining beta cell mass. This likely arises due to the fact that, as the beta cell population decreases, so too does the chemokine signal to which the immune cells respond. This means that the cells (on average) move into the islet at a slower rate, since their paths becomes more dominated by noise. If the cell lifespan is too short, the immune cells may die before entering the islet if there are insufficient beta cells generating a chemokine gradient.

### 4.3. Increasing the membrane degradation rate

Instead of reducing the basement membrane repair rate, we now increase the efficacy of the immune cells of degrading the membrane, setting λ_*m*_ = 0.2 and display the results in Figure 5. For comparison with the condition in which the encapsulating membrane is not repaired, we additionally plot results from this case in the same figure, as indicated.

As expected, increasing the rate of membrane degradation increases the rate of beta cell destruction and subsequently decreases the final remaining beta cell mass. Compared with the condition with no membrane repair, we see that the initial rate of beta cell destruction is greater in the case with high membrane degradation. However, as the insulitis process continues, this trend becomes reversed. Remarkably, across all cases varying the number of B cell and the immune cell lifespan, the time at which this occurs appears to be approximately the same. We also see that the final beta cell mass is lower for the condition with no repair than for that with high membrane degradation.

At the beginning of simulations, the ability of the T cells to degrade the basement membrane at a faster rate allows them to infiltrate the islet and subsequently kill the beta cells more quickly. Whilst the initial degradation of the membrane in the no repair condition is slower, the fact that “holes” in the encapsulating membrane caused by T cells are not repaired means that, at later times, immune cells can simply pass through these holes.

When the membrane can be repaired, the rate of immune cell infiltration is governed by the balance between how quickly it can be degraded and how quickly it can be repaired. As the chemokine signal across the peri-islet space becomes weaker when beta cells die, the average rate that T cells reach the basement membrane falls, since the strength of the signal is a contributory factor in determinining the transit time for a T cell toward the islet. As there are fewer T cells aggregrating at the islet membrane at any given time, the degradation of the membrane becomes slower, and hence the beta cells are destroyed at a slower rate as time progresses. At later times the arrival rate of T cells may be so slow that the membrane is repaired at the same rate as it is degraded, on average, and so islet infiltration by the immune cells will be halted.

These observations account for the slower rate of beta cell destruction and higher remaining beta cell mass for the high degradation condition compared to that with no membrane repair. This further highlights the importance of continual basement membrane repair to the slowing of insulitis.

### 4.4. Increasing the killing efficiency of the T cells

We now increase the killing rate of the T cells. To reflect the fact that we assume that activated T cells are responsible for most of the beta cell destruction, we achieve this by doubling the killing rate boost, setting Δκ = 1.8. The results for these experiments are displayed in Figure 6.

We observe that increasing the killing rate of the immune cells does not significantly affect the death rate of beta cells, either quantitatively or qualitatively. In all cases, we observe an increase in the beta cell mass destruction rate, but this is fairly minimal. The limited impact of increased killing rate can be understood by noting that, whilst the killing rate is an important factor in the overall insulitis process, this is dominated by transit time to and within the islet, as well as activation by B cells. Once inside the islet, T cells can interact with and kill beta cells but the total number of beta cells an individual T cell can destroy will be influenced more strongly by its remaining lifespan than on its killing rate. This highlights the need to incorporate the spatial aspects of the immune response and that high T cell killing rates do not necessarily significantly affect the progression of insulitis.

We comment that these observations at first seem at odds with previous results from the NOD mouse where the avidity of a T cell population is well-correlated with its pathogenic potential, and the progression of T1D occurs through avidity maturation (Amrani et al., 2000). Theoretical studies have supported the viewpoint that higher avidity CD8+ T cell populations lead to increased rates of beta cell destruction (Khadra et al., 2011) and that low avidity populations may have a protective effect against T1D development (Khadra et al., 2009). However, recent results in the NOD model have suggested that the highest avidity CD8+ cells do not escape thymocyte negative selection, and accordingly, the cells responsible for beta cell destruction in the murine pancreas may be regarded as those with “intermediate avidity” (Han et al., 2016). Furthermore, the paucity of data on the specificity of lymphocytes involved in human insulitis makes it difficult to propose quantitative statements about the effective killing potential of individual T cells. This is particularly difficult since the majority of the results regarding the avidity of human lymphocytes come from peripheral blood measurements and are not necessarily indicative of avidities within the pancreas (Reijonen et al., 2004; Standifer et al., 2009). Finally, the lifespan of a T cell that has successfully infiltrated an islet should still have a significant impact on the number of beta cells it can destroy, assuming its avidity is sufficiently high.

### 4.5. Increasing the rate of chemokine production

To reflect increases in the rate of chemokine production, we now double $\alpha_{C}^{1}$ and $\alpha_{C}^{2}$ simultaneously, setting $\alpha_{C}^{1}=1.0$ and $\alpha_{C}^{2}=20.0$. Note that, since we are assuming a quasi-steady state approximation for the chemokine, this simply adjusts the profile of the chemokine signal across our domain. The results for these simulations are depicted in Figure 7.

As for the situation with increased killing rates, we observe similar qualitative behavior to the case with lower chemokine production rates. However, in contrast to the former case, the quantitative differences between the beta cell death rate and final cell mass are much greater. This further highlights the contribution of cell movement in the overall inflammatory response.

### 4.6. Combining high chemokine signals and reduced membrane repair

**Figure 8:**
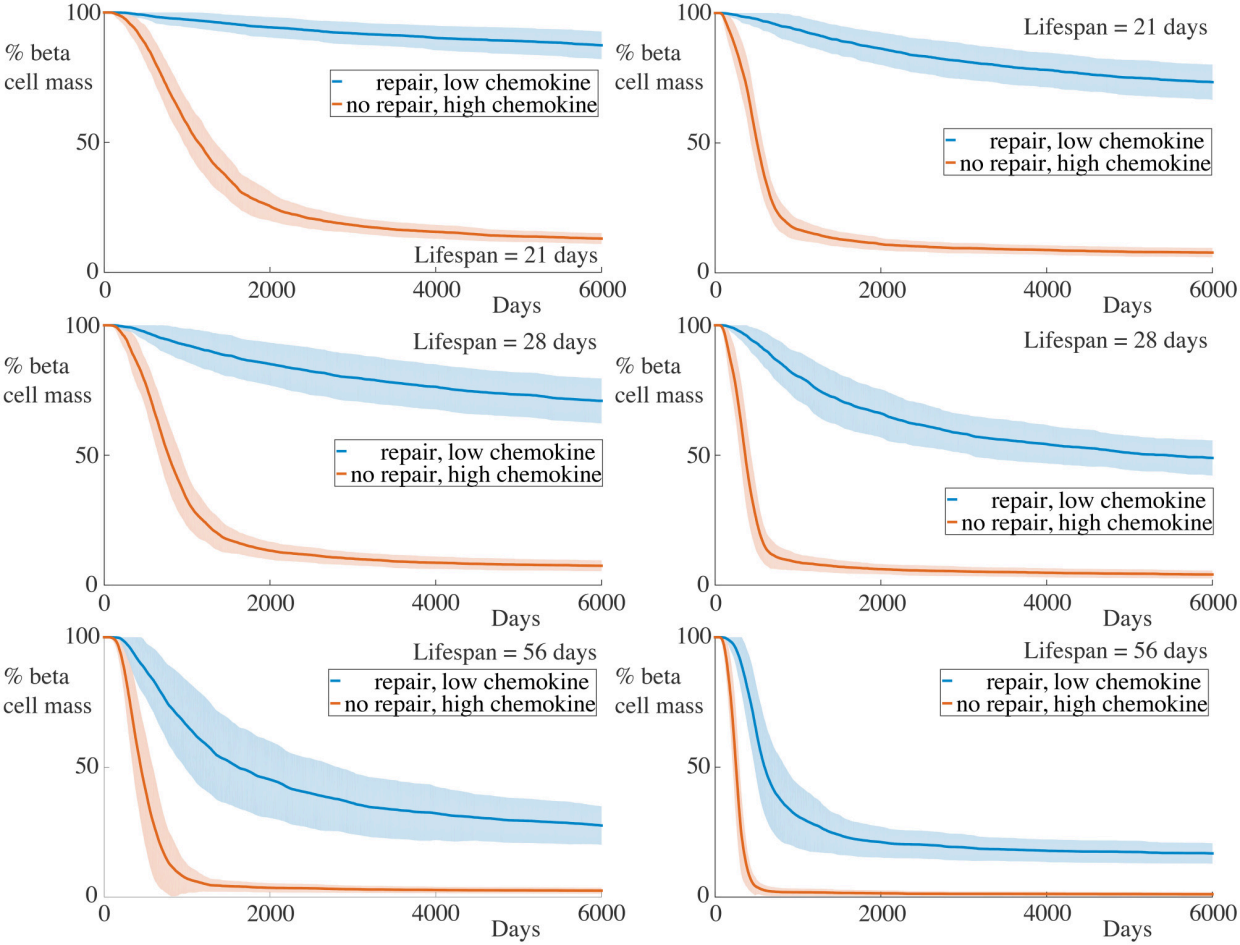
**Time-course of beta cell mass with high chemokine signals without basement membrane repair**. Each graph shows the mean ± SEM for 100 simulations. The left column shows results for simulations with 5 B cells whilst the right column shows those with 30 B cells. In all cases, the rate of beta cell loss is high and the remaining cell mass at the end of the experiments is low. Parameters are as in Figure 7 with α_*m*_ = 0.

In our final numerical experiment, whose results are shown in Figure 8, we simultaneously increase the strength of the chemokine signal, setting $\alpha_{C}^{1}=1.0,\alpha_{C}^{2}=20.0$, as in the previous subsection, and remove the membrane repair, setting α_*m*_ = 0. As expected, with these combined alterations, we now observe significantly high rates of beta cell destruction for all cases considered. Moreover, the final beta cell mass at the end of our simulations is below 20% of the original cell mass. For the cases with the longest lived immune cells, those having a lifespan of 56 days, we now see that essentially all of the beta cells have been destroyed within 1000 days.

### 4.7. Immune cell invasion profiles

**Figure 9:**
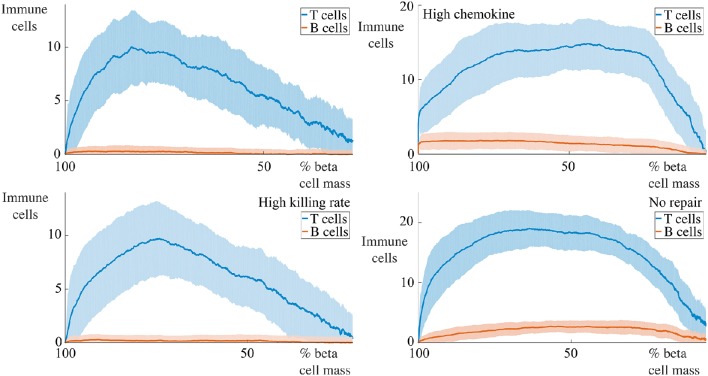
**Profiles of immune cell invasion dependent on the remaining beta cell mass in cases with 5 B cells**. Each graph shows the mean ± SEM for 100 simulations. We report the number of T and B cells within the islet in a number of conditions. The top-left panel shows results using the parameters in Table 1, the bottom-left uses these values with Δκ = 1.8, in the top-right, we set $\alpha_{C}^{1}=1.0$ and $\alpha_{C}^{2}=20.0$, and the bottom-right has α_*m*_ = 0.

**Figure 10:**
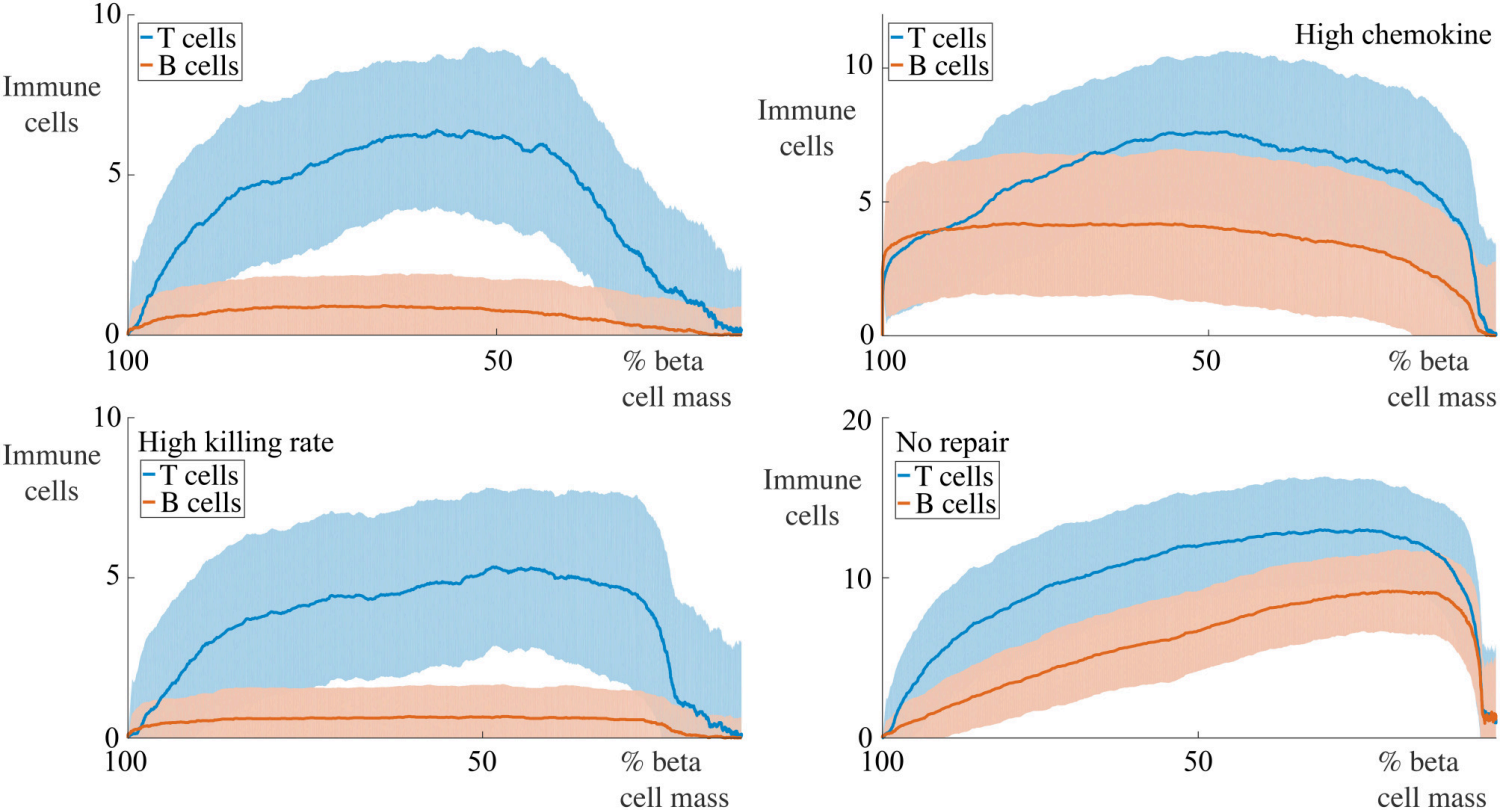
**Profiles of immune cell invasion dependent on the remaining beta cell mass in cases with 30 cells**. Each graph shows the mean ± SEM for 100 simulations. We report the number of T and B cells in the same conditions as displayed in Figure 9.

We now consider how the invasion of immune cells is dependent on the remaining beta cell mass and report the dependence of the number of immune cells within the islet (i.e., within the basement membrane) on the remaining beta cell mass. These results are shown in Figure 9 for the case with 5 B cells and in Figure 10 for the case with 30 B cells. The results for the case with high chemokine signals with no basement membrane repair, and for the case with increased membrane degradation are omitted, but are qualitatively similar to the case with no repair alone. In both figures, we only show results when the immune cell lifespan is 56 days.

For situations with 5 B cells, we observe two qualitative behaviors amongst the range of cases considered. Using the default parameters and those with high killing rates, we observe no infiltration of the islet by the B cells. The immune cell population undergoes an initial increase to a maximum value, followed by an approximately linear decay with the remaining beta cell mass. The behavior with high chemokine signals or no membrane repair does show islet invasion by the B cells. In these cases, the T cell population now both increases and decreases more gradually as a function of the beta cell mass, and attains a higher maximum value than in either of the two previous cases.

If we now increase the number of B cells to 30, we observe, in all cases, invasion of the islets by B cells and a qualitatively similar profile of T cell infiltration. With the default parameters, or those with high killing rates, the maximum value of invading T cells occurs when approximately 50% of the beta cell mass remains. In contrast to the T cell population, the number of invading B cells varies little with the beta cell mass. In the case with no membrane repair, this is no longer true, and we now find that the B cell profile follows the T cell profile and furthermore, that the maximum value of immune cell infiltration occurs at low values of the remaining beta cell mass.

### 4.8. Trial-to-trial variability and the role of space

For each of the scenarios listed, we report the standard error of the mean for all recorded values at each time point. In general, we observe that variability across our simulations for a given scenario is low, suggesting that the model behavior is robust and that noise does not significantly affect the intrinsic model dynamics. In particular, scenarios with either very high rates or very low rates of beta cell destruction exhibit very little variability. Variation is highest in scenarios that lead to intermediate rates of beta cell death, suggesting that the role of noise in such situations is much more significant in these conditions.

The results reported in Figures 3–10 reflect spatial averages that are taken over the peri-islet space. As such, they do not reflect the spatial component of the dynamics produced by the agent-based model. These effects are, in general, quite subtle. In particular, since we assume that activation of CD8+ cells by CD20+ cells requires them to be co-localized, the spatial arrangement of the immune cells will play an important role in determining the overall rate of beta cell destruction. The geometry in this initial version of the model has been chosen to be simplistic in nature. In future versions, the geometry will be adjusted to better match that of real islets as discussed in Section 5.1.1. One could imagine scenarios in which the routes taken by immune cells when migrating toward the islet could influence the average number of interactions with one another, and further, that these paths would be influenced by prominent vasculature near the islet.

The spatial configuration of the immune cells also plays a significant role in determining how quickly the basement membrane is degraded. Where many CD8+ cells accumulate at specific points along the periphery of the islet, we would expect high rates of membrane degradation. Conversely, if the cells are evenly distributed along the membrane, average degradation rates are likely to be low. Our results suggest that the dynamical processes governing the repair and destruction of the basement membrane play a key role in determining the overall rate of beta cell destruction and so it is clear that the spatial distribution of immune cells along this membrane will also be critical.

These spatial considerations are the key determinant for the degree of variability observed between trials, as reported in each of the figures. In the Supplementary Material, we provide animations of typical simulations in a subset of the scenarios listed to demonstrate the subtle role that these effects have on the insulitis process.

## 5. Discussion

In this manuscript, we have constructed a spatial, agent-based model of immune cell invasion of a prototypical islet of Langerhans. The agents in the model are immune and beta cells, and each obeys rules that broadly match the general behavior of these cell types. The model attempts to mimic a real islet, allowing for *in silico* experiments to be performed through model simulation. The agent-based framework allows both the spatial aspects of insulitis to be investigated, and avoids issues of applying ODE-based approaches to a system that has low cell numbers. It is intended that this will lay down the framework for more extensive spatio-temporal modeling of insulitis, with an ultimate aim to understand its core mechanisms and to devise strategies to slow or halt its progress.

**Figure 11:**
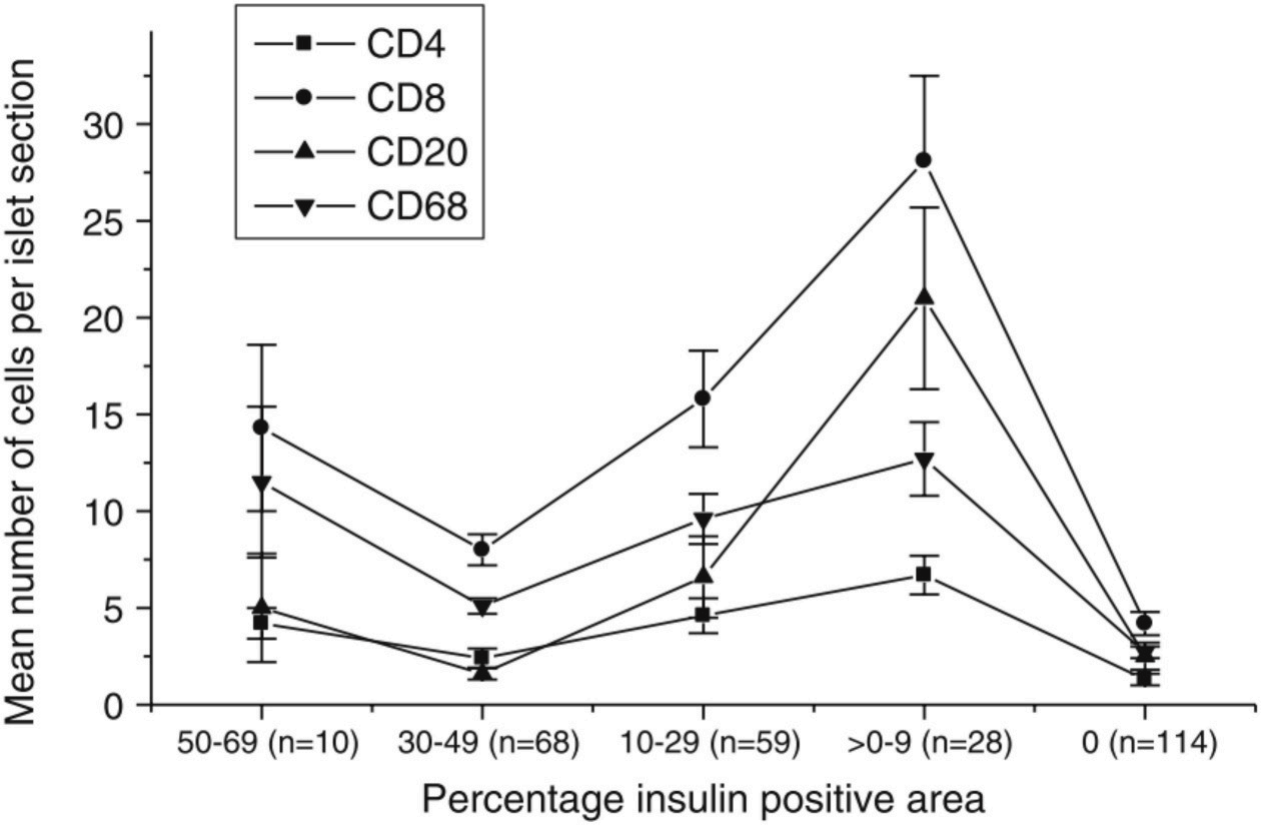
**Data showing the immune cell population against the percentage of the islet which is positive for insulin, as calculated via morphometry**. This percentage can be thought of as being representative of the residual beta cell mass. Each point is the mean ± SEM for the number of relevant immune cells detected in each islet section, averaged over 279 islets across 29 patients with T1D. The number of islets analyzed in each insulin-positive category is given in parenthesis. Permission to reprint this figure from Willcox et al. (2009) was granted by John Wiley and Sons (Licence number: 3971831129485; www.interscience.wiley.com).

Overall, the observed phenomena in the model are well matched to real data, which have been collected and analyzed from human patient samples (Willcox et al., 2009; Morgan et al., 2014), and are reported in Figure 11. We note that data for the pauci-immune phenotype have recently appeared in Leete et al. (2016), but since our model is primarily focussed on the hyper-immune phenotype, we highlight these results here. Results from the agent-based model have the same qualitative behavior as displayed by these data. This in turn provides evidence that the mechanisms included within our model are sufficient to capture qualitatively the key features of the inflammatory response. In particular, we note that the case with no membrane repair appears to provide the best qualitative match to the existing data, in which we see a rise in the number of infiltrating immune cells during disease progression, with a peak at around 10–20% of remaining beta cell mass. We note that scenarios with low overall rates of beta cell death are associated with low levels of B cell infiltration. This further supports the viewpoint that the CD20+ cells play an important role in determining the rate of beta cell destruction. By adding and removing mechanisms in a systematic way, we can probe what effect their inclusion or exclusion will have on the resulting dynamics and subsequent prognosis. It is important to note that, since we investigate the disease at the level of the islets, we can make predictions about how modifying certain cell properties will affect the inflammation of the islets, rather than considering whole body responses.

Accordingly, in the present study, we have performed several parameter studies to examine how the relative contributions of core processes affects insulitis. We have demonstrated that, under the assumptions used in constructing the model, an increased number of B cells in the peri-islet space gives rise to a faster destruction of the beta cells. This effect can be amplified by factoring in manipulations of other processes, such as the killing rate of the beta cells by T lymphocytes and the repair rate of the islet's basement membrane. Simply increasing the killing rate of the T cells did not have a significant impact of the rate of the beta cell destruction. However, increasing the strength of the chemokine signal to which the immune cells respond did show a pronounced increase in the death rate. These facts highlight the importance of the chemotactic process by which the immune cells enter the islet, find and kill the beta cells.

In scenarios in which the islet basement membrane is unable to repair itself, or is degraded more rapidly, we also see significant increases in the beta cell destruction. Moreover, we also find that the final beta cell mass at the end of our simulations is substantially depleted. This highlights the importance of this membrane as a barrier to the infiltrating immune cells. In particular, when chemokine signals are sufficiently high, if the membrane is incapable of being repaired, all of the beta cells may ultimately be killed, whereas in other situations, we find residual beta cells even after long simulation times (not shown). The importance of the basement membrane during insulitis in our model is in line with a recent study of this membrane in samples from human patients with T1D (Bogdani, 2016).

Upon examining the dependence of the immune cell invasion on the remaining beta cell mass, we find in most cases the profile of B cell infiltration is relatively flat. In cases with low B cell numbers, low chemokine signals and with a membrane that is repaired, there is essentially no B cell invasion. In situations with higher numbers of B cells, the beta cell mass at which the T cell invasion is maximal is shifted to lower values. In the case with no membrane repair, this maximal point is shifted to very low values of the remaining cell mass. Moreover, in this instance, we also see that the B cell invasion profile follows that of the T cells. This condition is most consistent with the human data reported in Willcox et al. (2009), suggesting that, for our model, low membrane repair rates are an important factor in the insulitis process.

The rise and subsequent decrease in T cell populations observed in our simulations is in accordance with experimental results of T1D specific autoimmune responses in the NOD model (Trudeau et al., 2003). These dynamics have been described as “waves” and have been explained mathematically by transitions through Hopf and homoclinic bifurcations in Mahaffy and Edelstein-Keshet (2007) and by transient bistability of the autoimmune state in Jaberi-Douraki et al. (2014b). Interestingly, in the latter of these two theoretical studies, inclusion of multiple clones of T cells with different avidities can give rise to multiple waves in the T cell dynamics, which can significantly impact the rate of overall beta cell destruction. This highlights potential avenues for intervention through the promotion of lower avidity T cell populations.

It should be noted that data on the specificities of T cells within human insulitic islets are rare, but that cells with varying antigen specificities may be present (Coppieters et al., 2012; Babon et al., 2016). Thus, a potential development of our model would be to include multiple clonal populations to explore these dynamics in the spatial context, particularly given the potential insights that may be applicable from the theoretical results in Mahaffy and Edelstein-Keshet (2007) and Jaberi-Douraki et al. (2014b).

Agent-based and lattice-based approaches are becoming increasingly popular in biological modeling. ODE based approaches, which have long been a linchpin of the mathematical biology community become ill fit for purpose when wishing to describe processes in which the number of agents is small. Considering stochastic variants of ODEs and partial differential equations (PDEs) models is one potential approach to account for the variability that arises from low cell numbers, but this too is only a good approximation when the number of cells is sufficiently high. Moreover, these approaches do not typically account for trial-to-trial variability that may be important for describing individual results. Note that, at various levels of description, systems of differential equations can still form part of agent-based models.

One of the attractive properties of ODE and PDE modeling when compared with agent-based simulations is the computational cost associated with the latter, whereas density (continuum) based methods typically are low dimensional in nature. Indeed, a significant barrier to the widespread use of agent- and lattice-based models in the past has been the requirement of significant computational power. In recent years, the technological advances in computing have started to erode this barrier. In addition, domain decomposition methods (Chen et al., 2007; Tapia and D'souza, 2011) and the ability to parallelise computations on large scales on commercially available graphics processing units (Chen et al., 2007; Tapia and D'souza, 2011; Harvey et al., 2015) has made agent-based simulations achievable universally.

Here, we have focussed on a specific type of agent-based model. This is based on a discrete particle formulation, where each cell is parameterized by a finite area and location. Broadly, agent-based models can be divided into lattice-based and lattice-free methods (Plank and Simpson, 2012). The discrete particle approach is lattice-free, meaning that cells are free to move anywhere in the domain. One limitation of this approach is that the cells are restricted to be described as circles; that is, their shape cannot be deformed. Immune cell deformation may play an important role when considering how cells pass through degraded parts of the basement membrane. In the current model formulation, cells must “wait” until the gaps in the membrane are sufficiently large before they can pass through.

An alternative to the discrete particle formulation is the lattice-based cellular Potts model. This modeling framework has been growing in popularity and has been applied to cell migration in a number of contexts, including vasculogenesis (Merks et al., 2006; Daub and Merks, 2013), morphogenesis (Chen et al., 2007; Marée et al., 2007) and wound healing (Scianna, 2015). Although the cellular Potts model is lattice-based, the cells within the framework can occupy many lattice sites and thus their shape, as well as their location, evolves over time. On a fine enough lattice, such models can look remarkably similar to the system they are approximating. However, with the increase in spatial resolution of the lattice comes an associated computational cost. It should be noted that there are several actively maintained packages for agent-based modeling, such as CHASTE (Mirams et al., 2013) and CompuCell3D (Swat et al., 2012).

One approach that combines the flexibility of lattice-free approaches together whilst allowing for cells to change shape is to treat points on the cell membrane as particles and resolve forces acting upon them, under the assumption that the cell remains bounded by its membrane (Elliott et al., 2012). However, the mathematical machinery and computing power required to embed such a description into our domain are prohibitive for our purposes. A “halfway” house could, in which the cells are treating as deformable ellipsoids, is another possible way of relaxing our assumption that cells are perfectly circular (Palsson and Othmer, 2000).

### 5.1. Future directions

The modeling framework described within this manuscript is not intended to be a fully comprehensive endpoint for the study of the spatio-temporal dynamics associated with insulitis. Instead, it is intended to be the first step to building a general virtual environment in which to simulate the invasion of islets during T1D. We will now highlight some potential areas for model development.

#### 5.1.1. Geometry

We have treated perhaps the simplest possible geometry that is reflective of the islet. The model is planar in nature, whereas real islets and immune cells are three dimensional structures. We have ignored the mechanical effects on the basement membrane: as beta cells are removed from the islet, the morphology of the islet itself may change (Brereton et al., 2014). There is currently no structure imposed upon the extra-islet space. Accurate modeling, for example, in which the vasculature is taken into account may constrain locations at which immune cells are allowed to enter the domain. Through the use of imaging techniques, it should be possible to build a more faithful representation of both the intra- and extra-islet space and include these in the model.

#### 5.1.2. Different cell types

The model could be developed to include more cell types, such as the macrophages that clear apoptotic beta cells, or different endocrine and exocrine cells which are not targeted by immune cells, but may slow their movement. There may be other cell-cell interactions that may be important during insulitis, for example the role of CD4+ cells in the activation of CD8+ cells (Castiglioni et al., 2005). Even restricting the model to the two immune cell types described in this manuscript, we could consider different clonal populations of the T cells (Khadra et al., 2011). One hypothesized strategy to slow the progression of insulitis is to promote the replication of a low avidity clone of T cells, that will out compete the higher avidity clones for space and resources, leaving only relatively passive T cells (Amrani et al., 2000; Khadra et al., 2009; Bluestone et al., 2015). Such a mechanism could be tested in our modeling framework.

#### 5.1.3. Cell proliferation

Currently, none of the cell types included in the model undergo cell division. In general, post-natal beta cell replication in humans is thought to occur at low rates, and we have disregarded it here. Similarly, immune cell replication in and around the islet is also assumed to be negligible. However, there is some evidence to suggest both that beta cell proliferation is enhanced during insulitis, at least during its early phases (Willcox et al., 2010; Dirice et al., 2014) and that immune cell replication also takes place within the islet itself (Graham et al., 2012) in the NOD model, though these findings have not been corroborated in human tissue. In particular, data collected from *ex vivo* human samples suggested that very few lymphocytes in the peri-islet space were positive for proliferative markers (Willcox et al., 2010). In order for the hypothesis of promotion of low avidity clones to be tested, the model would have to include cell division, since the action of long-lived memory cells is an integral part of that theory, however this may require coupling of the agent-based model with one representing the lymphatic system.

#### 5.1.4. Multiple islets

One of the striking features of insulitis is its heterogeneity, even within an individual patient. Islets near to one another can display different inflammatory profiles—insulitis in one islet thus does not imply insulitis in nearby islets. By extending our domain to include multiple islets, we can use the modeling framework to investigate under what scenarios this can occur. In particular, we can hope to identify what cellular interactions, on a broad scale, must occur for such heterogeneity in disease progression to be observed. One simple way to incorporate the influence of other islets in the current framework would be to include additional point sources of chemokine outside of the working domain. This would have subtle effects on the dynamics of cell movement, particularly when the number of beta cells becomes low.

#### 5.1.5. Membrane degradation

In the current model prescription, the membrane integrity is assumed to be compromised in a simple way by the action of nearby immune cells. This process is solely dependent on the proximity of the immune cells to the membrane. A more sophisticated approach might be to model the cells secreting proteases which diffuse freely and erode the membrane when they meet it. Whilst this is perhaps better fitted to the true processes that degrade the membrane, it is still not clear which cells are responsible for mediating this process. One potential route would thus be to consider the effects of having different immune cells responsible for this process within the modeling framework.

#### 5.1.6. Waxing and waning

One of the phenomena not accounted for in our description is the honeymoon period (Akirav et al., 2008). This is a phase lasting, in some cases, up to and beyond a year, typically immediately following diagnosis, in which the requirement for exogenous insulin is diminished. Though understanding of the mechanisms giving rise to this transient asymptomatic period is poor, one notable theoretical study demonstrated that this can occur naturally (in rare cases) when taking into account the role of endoplasmic reticulum stress and subsequent beta cell apoptosis (Jaberi-Douraki et al., 2014c). This study highlighted the possibility that elevations in the maximal unfolded protein response due to metabolic therapies could benefit patients by helping to meet metabolic demand, but despite this, they could not ultimately maintain a sufficient population of healthy beta cells to regulate blood glucose levels.

It is not clear whether the honeymoon period is reflective of events occurring at the level of individual islets, whether it is only present at the systemic level and whether it arises as a result of some innate periodicity present in either the immune system or in the beta cells. Modeling studies involving multiple islets may help to address some of these questions.

#### 5.1.7. Therapeutic intervention

Once a realistic geometry, motivated by real data, has been constructed, and the model sufficiently developed, an ultimate goal would be to test therapeutic interventions that target the pancreas specifically, such as the transplantation/implantation of encapsulated islets or populations of beta cells (Rickels et al., 2005; Robertson, 2010). We have discussed one hypothesis involving the promotion of low avidity T cell clones (Amrani et al., 2000; Khadra et al., 2009). One potential method for doing this is through the intravenous injection of *ex vivo*-selected and *ex vivo*-expanded autologous regulatory T cells, such as CD4+ cells (Bluestone et al., 2015), another is to incite the same expansion of regulatory T cells *in vivo* through the intravenous injection of nanoparticles coated with pMHC (Sugarman et al., 2013). Our modeling framework could easily be extended to include the effect of infusion of either the cells themselves or the nanoparticles to test the spatial aspects of these interventions.

#### 5.1.8. Cell-cell coupling

It is evident from the multiple sources of data that communication between immune cells is an important aspect of insulitis, though the exact processes by which these cells exchange information is unknown. One potential avenue of investigation is to assess specific cell interactions based on known immunological mechanisms (Huppa and Davis, 2003; Friedl et al., 2005). These can be incorporated into an agent-based model to see what the overall effects of these specific types of interaction are, with a view to identifying which signaling pathways are most likely to result in the behavior observed in patient data (McLennan et al., 2012).

#### 5.1.9. Comparison with animal models

Animal models, and in particular, the NOD mouse model have been extensively used to unravel potential causes and treatment options for human T1D. That true parallels between the disease in rodents and man may be fewer than is ideal, and the fact that treatments that work in mice are not as efficacious in humans (In't Veld, 2014) has led to questions about the suitability of animal models for human disease. From a modeling perspective, this opens up interesting questions. In particular, is it that the underlying mechanisms of insulitis truly are different between different species, or are some processes universal, albeit with potential different time courses and parameters? The former suggests that in order to find a cure for human T1D, only focus on the human condition is likely to yield fruitful results. However, the latter option suggests that, if relations between mice and men for those preserved pathways can be found, that the models can still provide vital insights. An *in silico* modeling framework seems like a natural place to address such a question as it allows quantitative probing of such relationships.

#### 5.1.10. Experimental design

One of the critical factors hindering progress in understanding T1D is the lack of human pancreatic samples from which to extract data. Moreover, these provide data only at one specific timepoint, so that very little is known about the time course of insulitis in humans.

One role for mathematical modeling is to identify avenues to explore to aid our understanding of disease mechanisms. The present study has suggested that the balance between repair and degradation of the peri-islet basement membrane surrounding the islets is a critical factor which determines the overall rate of beta cell destruction. Presently, little is known about the composition of this membrane in humans, though recent studies have identified its components in both healthy and infiltrated islets in the NOD mouse (Korpos et al., 2013). We thus feel that gaining an improved understanding of the structure of the human peri-islet basement membrane will significantly our improve understanding of insulitis.

Results from the agent-based model also suggest that the chemoattractant produced by the beta cells, and the lymphocytes' response to it is also important for governing disease progression. As such, *in vitro* experiments to assess and quantify the rate of immune cell migration to candidate chemokines would be useful to explore how immune cells initially reach the islet.

Finally, little is understood about the interaction between CD20+ and CD8+ cells during insulitis. The co-localization of these immune cell types in pancreatic samples suggest an interaction between the two (Huppa and Davis, 2003), and as such, we speculate that this could give rise to the activation of T cells. However, experiments have yet to confirm this for the immune cells involved in human T1D. Moreover, the specific dynamics of this process need to be better understood before the true role of the CD20+ cells in human insulitis can be revealed.

Overall, we believe that the proposed spatio-temporal framework has great potential as a tool to investigate insulitis in humans, and by focussing attention on the pancreas, it maximizes the use of the currently available human tissue data. We have identified a number of potential avenues in which the framework could be developed and a number of questions that could be addressed in doing so and we hope to continue in this line of research.

## Author contributions

All authors contributed to the initial design and subsequent development of the research project and model. KW developed code to run simulations and analyzed results from them. All authors drafted, edited and approved the submitted manuscript.

## Funding

This work was generously supported by the Wellcome Trust Institutional Strategic Support Award (WT105618MA). KT gratefully acknowledges the financial support of the EPSRC via grant EP/N014391/1. We are also pleased to acknowledge financial support from the European Unions Seventh Framework Programme PEVNET [FP7/2007-2013] under grant agreement number 261441 to NM. The participants of the PEVNET consortium are described at http://www.uta.fi/med/pevnet/publications.html. Additional support was from a JDRF Career Development Award (5-CDA-2014-221-A-N) to SR and project grant 15/0005156 from Diabetes UK (to NM and SR).

### Conflict of interest statement

The authors declare that the research was conducted in the absence of any commercial or financial relationships that could be construed as a potential conflict of interest.

## Supplementary material

Video S1 **Typical example of agent-based model simulation using parameters in Table 1, with immune cell lifespans of 28 days and ***B*** = 5**.

Video S2 **Typical example of agent-based model simulation using parameters in Table 1, with immune cell lifespans of 28 days and ***B*** = 30**.

Video S3 **Typical example of agent-based model simulation with no peri-islet membrane repair, as indicated in Section 4.2 with immune cell lifespans of 28 days, and ***B*** = 5**.

Video S4 **Typical example of agent-based model simulation with no peri-islet membrane repair, as indicated in Section 4.2 with immune cell lifespans of 28 days, and ***B*** = 30**.

Video S5 **Typical example of agent-based model simulation with high rates of peri-islet membrane degradation, as indicated in Section 4.3 with immune cell lifespans of 28 days, and ***B*** = 5**.

Video S6 **Typical example of agent-based model simulation with high rates of peri-islet membrane degradation, as indicated in Section 4.3 with immune cell lifespans of 28 days, and ***B*** = 30**.

Video S7 **Typical example of agent-based model simulation using in the high chemokine regime, as indicated in Section 4.5 with immune cell lifespans of 28 days, and ***B*** = 5**.

Video S8 **Typical example of agent-based model simulation using in the high chemokine regime, as indicated in Section 4.5 with immune cell lifespans of 28 days, and ***B*** = 30**.
